# Medical-grade honey as treatment for udder cleft dermatitis in dairy cows

**DOI:** 10.3168/jdsc.2025-0816

**Published:** 2025-08-06

**Authors:** Lisa Ekman, Susanna Holmberg, Josef Dahlberg

**Affiliations:** 1 Växa Sverige, 70288 Örebro, Sweden; 2 Department of Clinical Sciences, Swedish University of Agricultural Sciences, 75007 Uppsala, Sweden

## Abstract

Udder cleft dermatitis (UCD) is a common inflammatory dermatological condition of the udder skin in dairy cows, localized at the fore udder attachment or between the front udder halves. The severity of the condition can vary from superficial dermatitis to extensive wounds and different classification scales exist. To date, no effective curative treatment has been identified. Honey has been used in wound care for a long time due to its ability to promote healing and antimicrobial and anti-inflammatory properties. Thus, the aim of this pilot study was to investigate whether medical-grade honey could be as effective as zinc ointment in treating UCD. Fourteen cows with severe UCD (large crusts or open wound) were matched into pairs and then randomly assigned to treatment with medical-grade honey (n = 7) or zinc ointment (n = 7). Treatment continued for 8 wk, and UCD was graded at 3 different time points during the study. Treatment with medical-grade honey resulted in a higher improvement rate (42.9%) compared with zinc ointment (14.3%), although the result was not statistically significant. This study indicates that medical-grade honey could be an alternative treatment of severe UCD.

## Highlights

• Zinc ointment was compared with medical-grade honey to treat severe UCD.
• Medical-grade honey was well tolerated by cows and easy to apply to UCD lesions.
• A higher improvement rate was noted for medical-grade honey compared with zinc ointment.
• Medical-grade honey is a promising treatment alternative for severe UCD.

## Graphical Abstract

**Figure:**
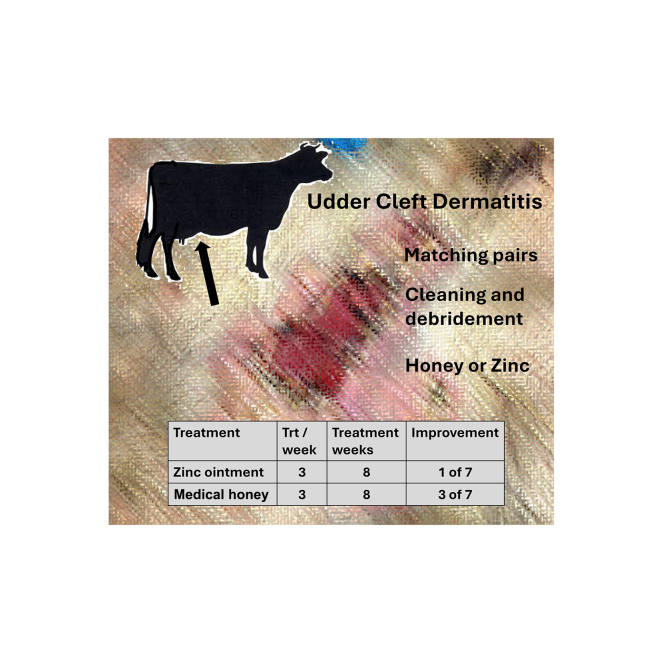
**Summary:** Udder cleft dermatitis (UCD) is an inflammatory dermatological condition of the skin localized at the fore udder attachment of dairy cows. The severity of the condition varies from superficial dermatitis to extensive wounds. The aim of this pilot study was to investigate whether medical-grade honey could be as effective as zinc ointment to treat severe UCD. Fourteen cows with severe UCD were matched into pairs and then randomly assigned treatment with medical-grade honey or zinc ointment. Cleaning and debridement were identical for both treatment groups. The cows were treated 3 times per week, and the treatment continued for 8 weeks. Treatment with medical-grade honey resulted in a higher improvement rate (3 of 7) compared with zinc ointment (1 of 7), although the result was not statistically significant. This study indicates that medical-grade honey can be an alternative treatment for severe UCD.

Udder cleft dermatitis (**UCD**) is a common dermatological condition affecting the cranial part of the udder in dairy cows. The condition can vary in size and severity, and different classification scales have been suggested based on macroscopic appearance (Olde Riekerink et al., 2014; Persson Waller et al., 2014; Bouma et al., 2016; Ekman et al., 2021). Although the different classification scales have different levels of categories, UCD is fundamentally categorized based on skin integrity, with mild UCD cases having intact skin and severe cases having an open wound. The prevalence of UCD has been examined in several studies and varies from 0% to 28% on a cow level and 0% to 62% at a within-herd level (Olde Riekerink et al., 2014; Persson Waller et al., 2014; Ekman et al., 2018; Groh et al., 2022). In a study from the Netherlands on a nonrandom selection of farms, the cow-level prevalence was as high as 52% (Bouma et al., 2016). Risk factors and the role of the microbiome and transcriptome have been examined to understand the occurrence and pathogenesis of the disease and are summarized in a review article by Vermeersch et al. (2025). Further, severe consequences such a embolic pneumonia (Millar et al., 2017) and renal amyloidosis (Capuzzello et al., 2024) have been reported to be associated with UCD. Spontaneous recovery from UCD has been found to occur in 38% to 61% of affected animals (Bouma et al., 2016; Ekman et al., 2021), but relapses are common, with reoccurring UCD lesions in affected animals.

Few effective treatment alternatives are available for UCD. A spray containing chelated zinc and copper has been evaluated, and Lammers et al. (2017) did see a positive effect on wound healing in a noncontrolled trial, whereas 2 other studies (Eikeland and Paulsen, 2018; Ekman et al., 2021) didn not see improved healing after 2 to 4 wk of treatment. The rationale of using chelated zinc and copper is the ability of zinc to simulate skin regeneration and copper's ability to reduce bacterial growth. Ekman et al. (2021) noticed improved wound healing in animals that underwent thorough cleaning but no additional treatment compared with control cows with untreated UCD. van Werven et al. (2018) examined 2 different treatment protocols in a randomized clinical trial; mild UCD cases were treated with an alcohol-free barrier film that was applied 3 times per week during 12 wk, and severe cases of UCD were treated with an alginogel consisting of hydrated alginate, polyethylene glycol, and an antimicrobial enzyme system that was applied once per day over 12 wk. For mild UCD cases, the treatment did not improve healing, but the treatment of severe UCD with enzyme alginogel showed 3.4 times more improvement compared with the control group. Thus, wound debridement and a favorable environment seem to be the key to improved healing.

Honey has a long history as a wound healing agent and has properties that are antimicrobial, anti-inflammatory, and stimulate wound healing (Cutting, 2007; Oryan et al., 2016). The antimicrobial properties of honey are partly due to the high osmolality and low availability of water. Honey also contains glucose oxidase, which converts glucose to hydrogen peroxide and gluconic acid under aerobic conditions. Hydrogen peroxidase has an antibacterial effect, and gluconic acid contributes to the low pH in honey (normally pH 3.2–4.5). The anti-inflammatory properties of honey are partly due to its antioxidant activity, which in turn is due to the presence of flavonoids, phenolic acids, ascorbic acids, tocopherols, and other compounds. Wound healing is stimulated by honey through different processes; the high osmolality stimulates a continuous flow of liquid from the wound and contribute to a moist environment, whereas hydrogen peroxide stimulates protease activity and glucose provides energy to somatic cells. It should be noted that botulinum spores can be present in honey, and for medical purposes, medical-grade honey (**MGH**) is recommended (Simon et al., 2009). Medical-grade honey has been used on human chronic wounds (Yapucu Güneş and Eser, 2007; Kamaratos et al., 2014) and proved to be more effective or equally effective as comparative treatments. In horses, MGH has been shown to improve healing in surgically treated lacerations (Mandel et al., 2020).

The aim of this pilot study was to compare MGH to a zinc-containing ointment as a treatment for severe UCD, using wound healing as a determining factor, with the hypothesis that MGH is as effective as zinc.

The study was performed at the Swedish Livestock Research Centre (Uppsala, Sweden) under the local ethical permit SLU-ID: Dnr: 5.8.18-06784/2020. Animals were recruited to the study by manually inspecting the front udder attachment of most the ∼270 lactating dairy cows. Eighteen cows with a suspected UCD were taken to a hoof trimming chute, where excess abdominal hair was removed and the front udder attachment was cleaned (using soap and water until visually clean), mechanically debrided, and patted dry with a paper towel. The UCD lesions were documented, measured, and categorized according to the 5-grade classification scale by Ekman et al. (2021). Briefly, scores of 3 to 5 are considered severe, with a score of 3 implying intact skin but crusts covering ≥5 × 5 cm, and higher scores implying an open wound and an total affected area that is smaller (score 4) or larger (score 5) than 5 × 5 cm. Fourteen cows fulfilled the criteria of severe UCD. To minimize the effect of confounders, the animals were matched into pairs and randomly assigned to treatments. Matching was based on UCD score, age, and DIM, in that particular order (Table 1). No information about UCD duration was available. Treatment consisted of cleaning, mechanical debridement, and covering the UCD lesion with a thin layer of MGH (L-Mesitran Soft, Theo Manufacturing, Maastricht, the Netherlands) or KRUSAN zinc ointment (Kruuse, Langeskov, Denmark). Zinc ointment was the currently used treatment for UCD lesions at the farm. Follow up treatment was performed by the barn staff or the research team in cubicles in the freestall barn on Mondays, Wednesdays, and Fridays for 8 wk. Before application of products, the lesions were cleaned and patted dry. Evaluation of wound healing was performed after 4 and 8 wk, after cleaning the lesion and with the animals in the trimming chute. At each evaluation, UCD lesions were scored and documented, and the collected data were transferred to a Microsoft Excel document. Statistical tests, *t*-test of equal means between groups before treatment, and a sign test for pairwise comparison of improvement of lesions after 8 wk were performed in PAST (Hammer et al., 2001) with significance level set to *P* = 0.05.

**Table 1:** Udder cleft dermatitis (UCD) score and cow information for 14 cows included in a randomized treatment study comparing medical-grade honey (MGH) and zinc ointment as treatment for UCD

Pair	Cow	Treatment group	Age in years (d 0)	DIM (d 0)	Grade UCD d 0	Grade UCD after 4 wk	Grade UCD after 8 wk
1	1	MGH	7.6	310	5	5	5
	2	Zinc	8.0	31	5	5	5
2	3	MGH	4.9	308	5	5	5
	4	Zinc	4.8	201	5	5	5
3	5	MGH	4.3	322	4	4	4
	6	Zinc	4.0	276	4	4	4
4	7	MGH	6.3	34	4	4	2
	8	Zinc	6.8	208	4	4	4
5	9	MGH	6.0	263	4	2	2
	10	Zinc	4.7	195	4	4	4
6	11	MGH	3.6	80	4	1	0
	12	Zinc	3.3	88	4	3	3
7	13	MGH	3.3	77	4	4	4
	14	Zinc	3.8	169	4	4	4
Total		Mean MGH	5.14^a^	199^a^	4.29^a^	3.57	3.14
		Mean zinc	5.06^a^	167^a^	4.29^a^	4.14	4.14

a Columns with the same superscript letter do not differ in *t*-test (*P* ≥ 0.05). Groupwise comparison of mean grade at 4 and 8 wk not performed due to non-numerical data.

Udder cleft dermatitis score, age, or DIM did not differ between the 2 treatment groups before first treatment, although cows in the zinc group on average were 33 d earlier into their lactation (Table 1). Of the 7 cows that were treated with MGH, 3 cows had a lower UCD score after the experimental period, of which one cow was completely healed. For the cows treated with zinc, 1 out of 7 had a lower score after the experimental period (Table 1). Udder cleft dermatitis lesion improvement was compared between the pairs of cows in a sign test but was not significant (*P* = 0.5).

Although no significant differences between treatment groups were noted in this study, more cows treated with honey improved and had a lower score after 8 wk, hinting that honey may be a successful treatment for cows with UCD. The low number of included animals is a clear limitation in this study, as well as the lack of a third group that only received cleaning and debridement, which would be desirable in future studies. None of the cows with UCD grade 5 improved during this study, and this is in line with previous studies showing that larger lesions have a smaller chance of healing (van Werven et al., 2018; Ekman et al., 2021). Because no information about duration or onset of UCD was available in this study, it is reasonable to assume that larger lesions are older and more chronic and are therefore more difficult to treat. The lack of information on UCD duration is a limitation, but simultaneously a probable on-farm scenario, as the location of UCD lesions often makes them go unnoticed. Due to the limited number of available cases with severe UCD at the Swedish Livestock Research Centre, the sample size and the statistical power are low. By matching the animals into pairs, some, but not all, confounding factors were balanced between the groups, giving higher priority to higher ranked risk factors. Because no prior information on expected treatment outcome was available, no pretreatment power analysis was conducted for this pilot study. Subjective observations that were made during the study indicate that both treatment protocols were tolerated well by the animals and that treatment performed in cubicles was feasible but not as thorough as in the trimming chute. Practically, the zinc ointment was easier to apply to open wounds but often stuck to the edges and dried, leaving a solid cake that was difficult to remove before next application. The honey, on the other hand, was more difficult to apply to open wounds but easier to remove during the following cleaning.

In conclusion, in this pilot study, MGH was compared with zinc ointment as treatment for severe UCD in dairy cows. It was well tolerated by the animals and easy to apply, but not significantly more effective. Medical-grade honey thus may be a promising alternative treatment for severe UCD.
